# Professor Matilde Leonardi, Fondazione IRCCS Istituto Neurologico Besta, Milan, Italy: Adapted Interview from the 12^th^ World Congress for NeuroRehabilitation (WCNR), Vienna, 2022

**DOI:** 10.25122/jml-2023-1017

**Published:** 2023-04

**Authors:** Stefana-Andrada Dobran, Alexandra Gherman

**Affiliations:** 1 RoNeuro Institute for Neurological Research and Diagnostic, Cluj-Napoca, Romania; 2 Sociology Department, Babes-Bolyai University, Cluj-Napoca, Romania

**Interviewer: Stefana-Andrada Dobran**

**Interviewee: Professor Matilde Leonardi**

Professor Matilde Leonardi is an Italian neurologist and pediatrician who currently serves as the Director of Neurology, Public Health, Disability Unit, and Coma Research Centre at the Carlo Besta Neurological Institute in Milan. She is a WHO expert and consultant on neurology, disability, ageing, and policy development, having research interests in disorders of consciousness, neurological aspects of ageing and disability, ICF and its biopsychosocial model, and burden of diseases.

Professor Leonardi is Neurology Ambassador for the ONE neurology network and a Fellow of the European Academy of Neurology (FEAN). She is also Chair of the EAN Communication Committee and a Presidium Member of the World Federation for NeuroRehabilitation Societies (WFNR), member of the WFNR Flying Faculty, and WFNR SIG Chair. Furthermore, she serves as co-chair of the WHO NeuroCOVID Forum group on essential neurological services for COVID-19 recoverees. In November 2011, she was appointed by the Vatican as a corresponding member of the Pontifical Academy for Life (*Pontificia Accademia Pro Vita*). Professor Leonardi is the coordinator of the Disability Management Project in Ukraine since March 2022 and in December 2022 was appointed by the Italian Government as Member of the National Committee of Bioethics.

**S.A.D.:** Hello, dear Professor Leonardi! We are here, in Vienna, for the 12^th^ World Congress for NeuroRehabilitation organised by the WFNR. What is your first-hand opinion of the event so far, and have you participated in any previous editions?

**M.L.:** I had the pleasure of that, I have been participating since the first edition 24 years ago. From the beginning, I’ve been following the growth of this important organization, and finally now, after three years, we meet again. It’s a sensation of joy to meet old friends, to know new friends, and to speak about the professional advancements that have been and are happening in the field of neurorehabilitation in these years. The World Congress is certainly putting together all the things, the personal and the professional elements – they are here. We are more than 1,100 people from many, many countries, sharing the same passion for neurorehabilitation and our work; so the atmosphere is very positive, and I think it is great to be here and to share with many other colleagues, from many other parts of the world, the pleasure of our profession and also the engagement that we have in our respective countries.

**S.A.D.:** What do you believe is the overarching perspective of this year’s congress?

**M.L.:** The idea is that rehabilitation is really changing a lot. There have been many things around the world that are framing rehabilitation in a different perspective; there are global initiatives such as in ten years reaching the sustainable development goal for health (SDG), in particular, the SDG 3 HEALTH – and rehabilitation is certainly a part of that, and there is Rehabilitation 2030 - the WHO Rehabilitation 2030 initiative draws attention to the profound unmet need for rehabilitation worldwide, and highlights the importance of strengthening health systems to provide rehabilitation. The initiative marks a new strategic approach for the global rehabilitation community by emphasizing that rehabilitation should be available for all the population and through all stages of the life course, rehabilitation should be integrated into all levels of health care and finally, and most importantly, that rehabilitation is an essential health service and crucial for achieving universal health coverage. Thus, this is a big global initiative of WHO, promoting the key idea that rehabilitation is not something that should be considered once all the rest is planned, it’s something that has to enter into the health programs as an essential health issue. The WFNR is really crucial in this, neurological patients are those suffering the highest burden worldwide, and as we are just finishing the Covid-19 pandemic, it is clear that during this pandemic, rehabilitation systems suffered due to outpatients closing or lockdowns restrictions so that rehabilitation could not be provided to many patients in many parts of the world, revealing how important it is to have rehabilitation clearly structured and embedded in the health system. I think we also have to state this – that wherever you are in the world, whatever age you are, rehabilitation is something that is good to help you recover your functioning and that is an essential service needed to reach universal health coverage. This is the overarching theme I think we have here, trying to reinstate rehabilitation as an essential health service. Ministries of health have to take into account rehabilitation as part of the things that can be offered to patients with chronic neurological disorders.

**S.A.D.:** How does a multidisciplinary team approach enhance neurorehabilitation?

**M.L.:** Neurorehabilitation has been evolving over the last 30 years. And it is certainly true that one person alone cannot respond to all the needs of a person who has a chronic condition. From a bio-psycho-social perspective, answering to the needs of people requires many people that do different things, and altogether they become a multidisciplinary team. Together with technology and assisted devices, that are one of the possible reasonable accommodations to be offered to patients. Multidisciplinarity means not only many people with the same mind but many minds for the same person. That’s the idea towards achieving and answering the needs. And it is much easier to do when you are many, able to use the same language than if you have to answer many questions alone, from a person.

**S.A.D.:** What is the importance of the patient outcome and follow-up in determining an individual outcome?

**M.L.:** More and more, it is clear that patients’ needs are driving the health demands. And patient’s needs can vary a lot; they are not only health-related needs, patients certainly have needs for good diagnosis, good care, and good treatment, but I think rehabilitation is going beyond all this and is going also in the life events. So, the patient-reported outcome measures are important because people, despite having a health condition, they want to have friends, they want to have a life, they want to work, they want to go to school, so rehabilitation has a role to bridge between health as well as other needs of a person. That is why it is important that patients are also able to tell their needs, but we, as health professionals, have to ask – ”What are your needs?”. So, it is an asking-and-answering paradigm that needs to be implemented.

**S.A.D.:** How can WFNR support the WHO’s Global Action Plan on Epilepsy and Other Neurological Disorders?

**M.L.:** In May 2022, it was signed as a key milestone for neurology worldwide. It has been approved by the World Health Assembly, the WHO Global Action Plan on Epilepsy and Other Neurological Disorders, WHO GAP, which provides a 10-year plan to countries to implement neurological care, but also to support brain health. And I think the WFNR and the EFNR in Europe, particularly, are in the position to, first of all, state what is brain health. Brain health is not the absence of the disease; brain health is all the possibilities that you have, despite your disease, to achieve a life that is full of satisfaction for what it is your need. More precisely WHO defines brain health as the state of brain functioning across cognitive, sensory, social-emotional, behavioural, and motor domains, allowing a person to realize their full potential over the life course, irrespective of the presence or absence of disorders. And in this sense, I think that all those dealing with neurology and neurorehabiliation are very much in the position to be advocates for brain health, with ministries as well as with many relevant stakeholders and that is what is happening mostly in many countries. There is a major role to play for all Global and Regional Scientific and Patients societies. In Europe, for example, the European Academy of Neurology is working on this – trying with its EAN Brain Health Strategy, to increase the awareness at ministries’ level showing that beyond diagnosis and care also prevention and promotion of brain health are feasible and that a lot can be done to prevent neurological disorders. For example, stroke can be prevented in many manners – promoting avoiding smoking, avoiding unhealthy diets and moving, and so on with all the things that we all know but we are never putting together and strongly connecting with brain health. I think it’s time to turn and make this “neurology revolution” that the Rehabilitation 2030 and the WHO GAP are asking all to do. I believe that this WFNR world congress is the turning point also for the WFNR that is becoming one of the world’s leading actors to speak about brain health and to promote brain health worldwide. What we are looking for is **brain health and neurology ambassadors**. I think everybody who is listening to this and is working in the field of neurology with neurological patients could become an ambassador for brain health in his or her country and could also promote, for example, the development of patient associations that can work together with health professionals to increase awareness, at a political level, about the importance of working for patients and with patients, and preventing and treating neurological diseases wherever possible. Prevention is possible, and together – good prevention and good care can increase the well-being and the quality of life of neurological patients worldwide. WFNR and EFNR are part of this global effort and we are at the world congress to affirm this all together!

**S.A.D.:** Thank you very much, Professor Leonardi!

**M.L.:** Thank you very much!

